# Light-Induced Clusterization of Gold Nanoparticles: A New Photo-Triggered Antibacterial against *E. coli* Proliferation

**DOI:** 10.3390/nano13040746

**Published:** 2023-02-16

**Authors:** Angela Candreva, Renata De Rose, Ida Daniela Perrotta, Alexa Guglielmelli, Massimo La Deda

**Affiliations:** 1 Department of Chemistry and Chemical Technologies, University of Calabria, 87036 Rende, Italy; 2 CNR-NANOTEC, Institute of Nanotechnology U.O.S, Cosenza, 87036 Rende, Italy; 3 Department of Biology, Ecology and Earth Sciences, Centre for Microscopy and Microanalysis (CM2), University of Calabria, 87036 Rende, Italy; 4 Department of Physics, NLHT-Lab, University of Calabria, 87036 Rende, Italy

**Keywords:** gold nanoparticles, light-induced clusterization, pathogen responsive, *E. coli* infection

## Abstract

Metallic nanoparticles show plasmon resonance phenomena when irradiated with electromagnetic radiation of a suitable wavelength, whose value depends on their composition, size, and shape. The damping of the surface electron oscillation causes a release of heat, which causes a large increase in local temperature. Furthermore, this increase is enhanced when nanoparticle aggregation phenomena occur. Local temperature increase is extensively exploited in photothermal therapy, where light is used to induce cellular damage. To activate the plasmon in the visible range, we synthesized 50 nm diameter spherical gold nanoparticles (AuNP) coated with polyethylene glycol and administered them to an *E. coli* culture. The experiments were carried out, at different gold nanoparticle concentrations, in the dark and under irradiation. In both cases, the nanoparticles penetrated the bacterial wall, but a different toxic effect was observed; while in the dark we observed an inhibition of bacterial growth of 46%, at the same concentration, under irradiation, we observed a bactericidal effect (99% growth inhibition). Photothermal measurements and SEM observations allowed us to conclude that the extraordinary effect is due to the formation, at low concentrations, of a light-induced cluster of gold nanoparticles, which does not form in the absence of bacteria, leading us to the conclusion that the bacterium wall catalyzes the formation of these clusters which are ultimately responsible for the significant increase in the measured temperature and cause of the bactericidal effect. This photothermal effect is achieved by low-power irradiation and only in the presence of the pathogen: in its absence, the lack of gold nanoparticles clustering does not lead to any phototoxic effect. Therefore, it may represent a proof of concept of an innovative nanoscale pathogen responsive system against bacterial infections.

## 1. Introduction

Gold nanoparticles are widely used in the manufacture of numerous products, from electronics to biomedical devices [1,2,3,4,5,6,7,8,9]. In particular, they have raised high interest in cell biology and biomedicine due to their unique chemical, optical and electronic properties that result from their minute size [10,11,12,13]. Interestingly, when light interacts with gold nanoparticles, it is both scattered and absorbed causing a surface plasmon excitation and the resulting spectral shape of the absorbed radiation depends on many factors such as the size, shape, composition, and environment of the nanoparticles [14,15,16,17,18,19,20]. Over the years, scientists around the world have been experimenting with the synthesis of metal nanoparticles of different shapes and sizes. This is because the properties of nanoparticles depend on their nanostructure. By changing the nanostructural properties, the plasmonic properties change accordingly. Great interest has been aroused in the gold nanoparticles, which have a plasmon band that falls in the visible range, but can also move towards the wavelengths of NIR [21,22,23]. In addition, the surface of gold nanoparticles can be easily functionalized with amino groups or thiol groups [24], and still biomolecules such as DNA and proteins are used as covering agents of gold nanoparticles [25]. The ease of characterization of nanoparticles has also contributed to increased interest in the “nano world” [26,27].

A key aspect in uncovering more new biomedical applications of gold nanoparticles is their cellular uptake [28,29]. Despite numerous studies in this field, the current understanding of the factors influencing the cellular internalization of nanoparticles remains very limited. It is accepted that commonly used gold nanoparticles are able to cross cell membranes, usually via endocytic pathways; however, the effectiveness of absorption depends on the charge, as well as the size, shape, and surface chemistry of the nanoparticles [29,30,31,32,33,34,35,36,37,38]. Bulk gold is considered to be biologically inert; on the other hand, at nanoscale size, gold has different attributes due its surface plasmon resonance excitation features [39]. It is important to underline that, as the nanotechnology field continues to develop, several studies in understanding the size- and shape-dependent toxicity of gold nanomaterials are being carried out. These studies are increasingly emphasizing how different morphologies of the nanoparticles have a different impact on organisms and on the consequent applications [40,41,42,43]. Several studies have been conducted on clusterization of nanoparticles during their interaction with living cells. The result is that the nanoparticle cluster greatly enhances diagnostic sensitivity and therapeutic effectiveness compared to individual nanoparticles [44].

Gold nanoparticles (AuNP) are appealing photothermal candidates because they show efficient local heating upon excitation of surface plasmon oscillations [45,46,47]. The strong absorption, efficient light/heat conversion, and high photostability, contribute to arousing increasing interest in the photothermal applications of gold nanoparticles that permit a directional control of the incident radiation on the administration region of the these phototransducers, resulting in localized heat transfer to the surrounding environment [28,48,49,50]. When discussing the photothermal activity of gold nanoparticles, several important parameters are implicitly considered: the wavelength of the laser, that should be matched with the peak of the plasmonic band of the used nanoparticles [51], the power of the laser, and the nanoparticle’s size. In particular, the higher the power of the laser, the higher the temperature increase [5,46,52,53]. Furthermore, by increasing the size of the nanoparticles, the temperature increases as well [17]. However, a high-power laser can itself cause damage to the cellular environment, and it is not possible to administer nanoparticles with a large size [54].

Herein, we show the interaction between gold nanoparticles and bacterial populations [55,56,57], i.e., *Escherichia coli* [58,59,60,61], in dark or light conditions [62,63,64]. In fact, recently, gold nanoparticles have also gained interest for their antibacterial properties against different microorganisms [65]. We prepared 50 nm-diameter gold nanospheres, covered with thiolate polyethylene glycol (PEG-SH), well known to not have significant antibacterial effects [36]. From an accurate study performed by the use of electron microscopies, it was possible to observe the uptake of nanoparticles within bacterial cells as well as the pleomorphism (rough surface) and shrinkage in size because of increased cell death [61,66,67,68,69,70] An antibacterial test, performed by varying AuNP concentration (from 0.26 to 3.54 µg/mL), clearly indicates that *E. coli* exposure to gold nanoparticles inhibits bacterial growth, and that this inhibition, directly proportional to the nanoparticle concentration, is mainly observed in light conditions. We administered the light to the bacteria simultaneously with the dosage of the nanoparticles, and this caused the fast aggregation of nanoparticles, induced by light [71], on the bacterial surface. According to our previous studies [17,18], the clusters have a greater photothermal effect than single nanoparticles, and this was confirmed by the total bacterial inhibition growth. Since this clustering occurs only in the presence of bacteria, this system may represent a proof-of-concept of an innovative nanoscale pathogen responsive system against bacterial infections. To the best of our knowledge, we are proposing the first example of a system able to activate only in the presence of the bacterial cells, and to have a bactericidal effect even at low concentrations. In particular, it is important to underline that in this work several limitations have been overcome, in fact, a low-power laser and small-sized gold nanoparticles have been employed obtaining, as the result, a complete inhibition of bacterial *E. coli* growth.

## 2. Materials and Methods

*Chemicals*. All chemicals were purchased from Sigma-Aldrich (Schnelldorf, Germany) (highest purity grade available) and used as received. Tetrachloroauric acid trihydrate (HAuCl_4_·H_2_O, ≥99.9%), sodium citrate C_6_H_5_Na_3_O_7_·2H₂O (99%), Milli-Q water (resistivity 18.2 MΩ·cm at 25 °C) were used in all experiments. All glassware was washed with aqua regia, rinsed with water, sonicated threefold for 3 min with Milli-Q water, and dried before use.

Sterile tissue culture plates of polystyrene, 6-well, 35mm, non-treated Biofil. *E. coli* (DSM 1576 Medium 1) from DSMZ (German Collection of Microorganisms and Cell Cultures, Braunschweig).

*Exponential growth phase*. *E. coli* solution was diluted (1:100 *v*/*v*) in 20 mL of fresh DMS Medium 1 to restart the cell cycle and after 3 h of incubation at 37 °C the cells were synchronized at the log phase of the growth curve, featured with the optical density at 600 nm of 0.4–0.6. At this OD value, the cells divide and the growth rate is constant and the cells are in exponential growth phase.

*Gold spheres covered with sodium citrate (AuNS@Citrate)*. *Seed solution*. A water solution of sodium citrate (6.0 E-2 M) was stirred vigorously and heated until the boiling temperature. At this point, 1 mL of HAuCl_4_ 0.025 E-3 M was added. Immediately, the reaction was cooled to 90 °C. The decrease in the temperature provides the inhibition of a new nucleation, favoring the consequent overgrowth of the seeds. Sodium citrate has two functions: it reduces Au (III) to Au (0) and coats the seed to prevent their aggregation. Within 10 min, the color of the solution changed from yellow to bluish grey and then to soft pink. *Growth solution*. After temperature stabilized to 90 °C, 1 mL of sodium citrate (0.060 E-2 M) and 1 mL of a HAuCl_4_ solution (0.025 E-3 M) were consecutively added. By repeating this process various times, it was possible to obtain citrate-coated gold nanospheres with an increasing diameter [17,72]. To accelerate the process, the sample was diluted by extracting a 55 mL aliquot and adding 53 mL of hot distilled water to it, followed by 2 mL of 0.06 E-3 M sodium citrate water solution. When the temperature was stabilized at 90 °C, 1 mL of HAuCl_4_ was added. By repeating the process six times, nanospheres with 50 nm diameter were obtained, characterized by UV–Vis absorption spectroscopy and TEM.

**Figure 1:**
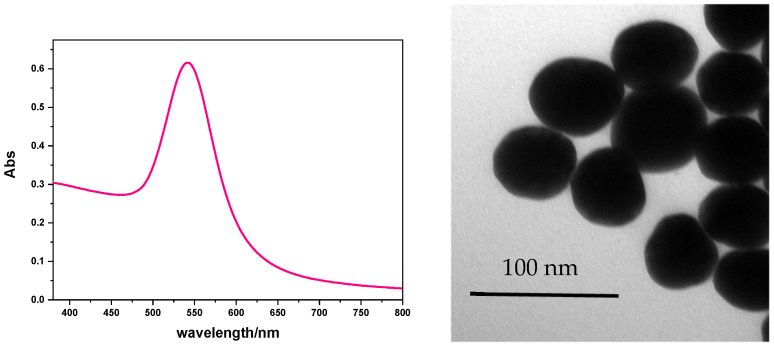
UV–Vis extinction spectrum and TEM image of AuNS@PEG-SH.

*Gold spheres covered with thiolate polyethylene glycol (AuNS@PEG-SH)*. A water solution of PEG-SH was prepared (30 mg in 1 mL) and added to 25 mL of 5 E-4 M water-dispersed AuNS@Citrate. The sample was left under stirring overnight, and then purified by unlinked PEG-SH (three centrifuge cycles, 600 rpm). The solid residue was dissolved in water (Figure 1) [15].

*Preparation of microorganism suspension*. The antibacterial potential of synthesized AuNPs with and without irradiation was tested against one human pathogen bacterial strain: *E. coli* (DSM 1576 Medium 1), as a model for Gram-negative bacteria. The lyophilized microorganisms were pre-cultured aerobically, with shaking in 50 mL of DSM Medium 1 for *E. coli* for 24 h at 37 °C, and were maintained on nutrient agar NA slants (0.5% beef extract, 1% peptone, 0.5% NaCl, and 1.5% agar). The organisms were stored at 4 °C and subcultured at regular intervals of 30 days to maintain the cell viability. The bacterium was transferred from stored slants at 4 °C to 10 mL of nutrient broth (meat extract, peptone, NaCl), and cultivated overnight at 37 °C. Considering that, for *E. coli*, an OD600 nm of 0.1 corresponds to a concentration of approx. 10^8^ cells/mL, for experiments, the bacterial cultures were diluted in sterile PBS (phosphate-buffered saline) to obtain a microorganism suspension of about 10^4^ cells/mL. To evaluate the antibacterial performances of gold nanoparticles against *E. coli* and of their photothermal bactericidal property, we used the growth inhibition assay. This method determines the number of viable bacteria, colony-forming units (CFU/mL) after 24 h of contact between microorganism and AuNPs, in the dark and irradiated. The bacterial suspension of 2 × 10^4^ cells, in the exponential phase of growth, were added to the gold nanoparticles diluted in the growth DSM Medium 1 (1:500) to reach four final gold nanoparticle concentrations: 0.26, 0.39, 1.56, 3.54 µg/mL, in a final volume of 2ml, and placed in petri wells of 35 mm. A duplicate of these samples was irradiated with a green light source for 5 min. In addition, the bacterial strain sample without nanoparticles was used as negative control. All petri wells were incubated at 35 °C for 24 h with 95% humidity and were shaken at 200 rpm on a stirrer plate (Orbital Shaker, PSU-10i, Grant-bio). Cell division occurs slowly because the growth medium is very diluted.

All samples were collected and 100 µL of appropriate dilution was spread over the surface of the nutritive agar using a sterile bent plastic rod. After incubation at 35 °C for 24 h, the number of CFU was evaluated. Each experiment was performed in duplicate and repeated 3 times. The inhibitory effect was calculated using the following formula:

Percent inhibition = 1 − T/C × 100

where T is the CFU/mL of the test sample after 24 h, and C is the CFU/mL of the control after 24 h [73,74].

Electron microscopy (TEM and SEM), dynamic light scattering (DLS), photophysical and photothermal measurements

*Transmission electron microscopy (TEM)*. The size and shape of gold nanoparticles were characterized using a transmission electron microscope (TEM). Samples for TEM were prepared by depositing a drop of a diluted solution on formvar/carbon-film-coated 300 mesh copper grids for 15 min. This process was followed by the removal of extra solution using blotting paper. After that, the grids were allowed to dry prior to measurement. The analysis was carried out on a JEOL JEM-1400 Plus transmission electron microscope at an operating voltage of 80 kV [75].

*Scanning electron microscopy (SEM)*. SEM analysis was carried out to investigate the uptake and effects of AuNP on bacterial cell morphology. Briefly, samples were fixed with 3% glutaraldehyde for 2 h and dehydrated with a graded series of ethanol solutions (50%, 60%, 70%, 80%, 90%, 99%, and anhydrous ethanol) for 10 min each. Prior to observation, specimens were coated with graphene films and finally viewed under a scanning electron microscope ZEISS Crossbeam 350 operating at 10.00 kV. Both secondary electrons (SE) and backscattered electrons (BSE) images were simultaneously acquired and compared. Acquisition by SE revealed topographic information with excellent resolution. With the use of BSE, the inorganic AuNP and the organic structures were distinguished by virtue of their different atomic number. This atomic number sensitivity creates contrast in the image where the inorganic NPs (with high atomic number) appear as bright spots, while the organic structures (bacterial cells) with low atomic number appear darker. This enables the ready visualization of inorganic NPs and their location both outside and/or inside the cells.

The analysis of interactions of gold nanoparticles with bacteria and their morphological effects requires high resolution imaging techniques due the extremely small size of the plasmonic metal nanoparticles. Nanoparticles are not individually distinguishable with conventional optical microscopy, since their size is below the resolution limit. Thanks to its high resolution, transmission electron microscopy (TEM) has proven to be a powerful tool that allows observation of nanoparticles inside the cells, and is widely used for the analysis of nanoparticle uptake and relationships with cell and tissue components [76]. However, sample preparation can be a rather challenging, laborious, and/or time-consuming process, and the obtained information for each sample is limited to the thickness of the cell slices [77]. A new generation of high-resolution SEM (HRSEM) provides less limitation, with a final image resolution better than 1 nm, that makes it possible to analyze the interactions/uptake of metallic nanoparticles by cells. HRSEM benefits from the rapid (but accurate) method for the sample preparation and the higher depth-of-field imaging, thereby providing detailed information on the 3D morphological organization of cells. Using this technique, it is possible to visualize the interaction of NPs with the cell membrane and map their 3D distribution.

*Dynamic light scattering (DLS).* Size distribution of nanoparticles was measured by dynamic light scattering, by using a Zetasizer Nano S from Malvern Instruments (632.8 nm, 4 mW HeNe gas laser, avalanche photodiode detector, 175° detection). The measurements were performed in triplicate at 25 °C. AuNS@PEG in water and AuNS@PEG in PBS were characterized. The results are showed in Figure S1 (Supplementary Material).

*Photophysical characterization.* Perkin Elmer Lambda 900 spectrophotometer was employed to obtain the absorption spectra [78,79,80]. An amount of 3 mL of the nanoparticle dispersion was transferred from the reaction flask to a quartz cuvette to carry out the measurement.

*Photothermal characterization.* Solutions were irradiated, within a customized thermo-optical setup, by using a CW laser source (gem532; Laser Quantum, Stockport, UK), emitting at 532 nm in the high-absorption plasmonic band of the investigated AuNPs. The laser beam acted perpendicularly (from the top) to the air/solution interface, using three mirrors, in the central part of a quartz cuvette. A high-resolution thermal camera was used to map and quantify the temperature increase of the AuNPs solutions under top-pumping laser excitation. The IR thermoimages were recorded by ThermoCamera FLIR (A655sc), providing thermal images with 640 × 480 pixels, with an accuracy of ±2 °C.

## 3. Results

The extinction spectrum in Figure 1 shows a 540 nm band due to the plasmonic resonance of 50 nm- diameter AuNS@PEG-SH, while the TEM image confirms the shape and size of synthetized gold nanoparticles.

AuNS@PEG-SH were administered to *E. coli* culture, at different concentrations, in dark or light conditions. The results of cell inhibition growth are shown in Table 1. In Table S1 (Supplementary Material), for more clarity, the results of the original experiments with counting colonies are also reported.

**Figure 2:**
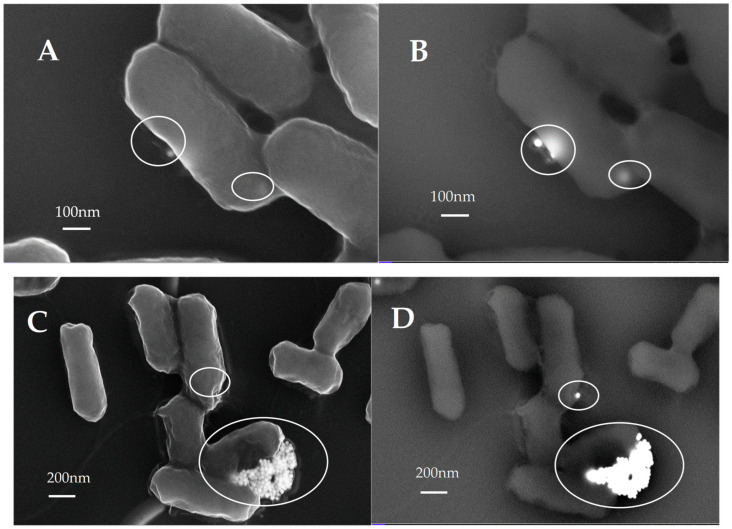
Secondary electrons (on the left) and backscattered electrons (on the right) paired images demonstrating the uptake of AuNP (3.54 µg/mL) in both non-irradiated (**A**,**B**) and irradiated (**C**,**D**) *E. coli* cells. Irradiated *E. coli* cells show greater pleomorphism (rough surface) and shrinkage in size because of increased cell death. Large aggregates of AuNP adhere onto the cell surface.

According to these results, in the dark, the administration of gold nanoparticles to *E. coli* has no detectable effect at low concentrations (0.26 and 0.39 µg/mL), while at the concentration of 1.56 µg/mL there is a growth inhibition of 15%, which reaches a greater bacteriostatic effect at the concentration value of 3.54 µg/mL, causing a growth inhibition of 46%. These results show that the MIC (minimum inhibitory concentration) of AuNS@PEG-SH in reference to *E. coli* culture is in the range 0.39–1.56 µg/µL. This behavior is due to the toxic effect exerted by the gold nanoparticles: due to their nano size, they penetrate cells, causing cytotoxic damage [81,82,83]. According to SEM images (Figure 2A,B), SE and BSE paired images demonstrate the uptake of AuNPs in non-irradiated *E. coli* cells.

To activate plasmon resonance, we irradiated the bacterial cultures by using a 532 nm laser source (i.e., at a wavelength matching the nanoparticles plasmonic band) with a soft power of 60 mW. The results reported in Table 1 show that the growth inhibition is equal to 53%, while at the same concentration in the dark the value was 15%; it increases as the concentration of nanoparticles increases, reaching the remarkable value of 99% when the concentration of gold nanoparticles is equal to 3.54 µg/mL (while this value measured in the dark was 46%). This exceptional inhibition value, that corresponds to a bactericidal effect, is attributed to the cytotoxic effect, due the gold nanoparticles’ uptake, combined with the photothermal one (Figure 2C,D). According to the literature, a bactericidal effect is obtained when the radiation induces a temperature increase over 60 °C [83,84]. By examining the SEM images of Figure 2C,D, a formation of gold nanoparticles clusters is clearly visible, which we assume to be responsible for the bactericidal photothermal effect.

Under the same conditions, an aqueous solution of AuNS@PEG-SH at a concentration of 3.54 µg/mL (i.e., the concentration inducing a bactericidal effect) was irradiated, achieving a temperature increase of 2 °C (Figure S2 in Supplementary Information). This slight increase in temperature, due to the irradiation of the gold nanoparticles, remained modest also by increasing the concentration of the nanoparticles: in a saturated solution, the maximum measured value was 11.6 °C (Figure S3 in Supplementary Information). Interestingly, in these aqueous solutions of AuNS@PEG-SH, cluster formation has never been observed. The only way to measure a noticeable rise in temperature was to increase the laser power (1500 mW). Indeed, by irradiating the gold nanoparticle solution with the concentration showing a bactericidal effect (i.e., 3.54 µg/mL), we measured a temperature increase of 26 °C (Figure S4 in Supplementary Information), while by irradiating the saturated solution, it showed a temperature increase of 35.4 °C (Figure S5 in Supplementary Information); in the latter case, we observed the formation of nanoparticle clusters. In fact, within seconds, a layer of gold appeared on the surface of the colloidal solution under irradiation. Upon interrupting the irradiation and shaking the sample, the layer of gold disappeared; this phenomenon, induced by high-power laser irradiation, is widely reported in the literature [44,71,85,86].

## 4. Discussion

The photothermal effect was studied both in the dispersion of nanoparticles alone (at two different concentrations, i.e., 3.54 µg/mL and saturated solution) and in presence of bacteria (at the 3.54 µg/mL concentration of nanoparticles). In the colloidal dispersion of nanoparticles alone, at the 3.54 µg/mL concentration, a relevant photothermal effect was not observed, while this effect was detected in the saturated solution, where clusters formation was observed. This leads to the conclusion that the clusters are responsible for the photothermal effect.

In the bacterial cultures that showed significant growth inhibition, clusters of gold nanoparticles were formed even at low concentrations (3.54 µg/mL) and with a low-power laser (60 mW). Since these clusters are formed, at this concentration, only in the presence of bacterial cells, we suppose that it is the bacterial wall that favors the aggregation of gold nanoparticles, once irradiated. It is precisely this cluster the effective photothermal tool, determining an increase of the local temperature, responsible of the observed bacterial growth inhibition and to the final cell death, as displayed in Figure 2C,D, where irradiated *E. coli* cells exhibit greater pleomorphism (rough surface) and shrink in size caused by cell death.

To obtain a photothermal effect, mediated by gold nanoparticles, it is necessary to have two requirements: a high-power light source and nanoparticles of considerable size. These two necessary aspects have disadvantages: the use of high-power lasers can itself be harmful to the cellular environment in many ways; the use of large nanoparticles makes administration difficult. In this work we have tried to overcome these two limitations by using a low-power laser and small size gold nanoparticles. A complete inhibition of bacterial growth was observed, due to the fact that the low-power laser induced the formation of gold nanoparticle clusters of such dimensions that are able to induce, under irradiation, a temperature increase capable of obtaining a bactericidal effect [17]. This fact, which under the same experimental conditions was not observed in the absence of bacteria, is due to a sort of catalytic effect exerted by the bacterial wall in the formation of the clusters, as can be clearly seen from the SEM images.

## 5. Conclusions

Local temperature increase is extensively exploited in photothermal therapy, where light is used to induce cellular damage. Gold nanoparticles, showing plasmon resonance phenomena when irradiated with electromagnetic radiation, are able to induce relevant temperature rises mainly dependent on their size.

In this work we present a proof-of-concept nano-sized system for a photothermal treatment of an *E. coli* culture. We synthesized 50 nm-diameter gold nanospheres covered with thiolate polyethylene glycol, AuNS@PEG-SH, and administered these nanoparticles in water solutions at different concentrations, to a bacterial culture, observing cell growth in the dark or under light irradiation.

Previously, we have measured the temperature values of the AuNP solutions under high-power (1500 Mw) laser irradiation, measuring a temperature value of 54.4 °C, while, as expected, low-power laser source (60 Mw) causes a negligible temperature rise; in both cases the values depend directly on the concentration of the solutions.

Bacterial treatments with AuNS@PEG-SH aqueous solutions show no growth inhibition at low concentration, while at 1.56 µg/mL and 3.54 µg/mL, we observe, in the dark, a bacteriostatic effect (46% of growth inhibition at a concentration of 3.54 µg/mL). SEM images show an uptake of nanoparticles in the bacteria cells, responsible for the observed bacterial inhibition growth.

To irradiate the bacteria, it was preferred to use a soft-power laser (i.e., 60 mW) in order to have no harmful effects due to the power of the source alone. Under these conditions, we observed an AuNP concentration-dependent inhibition of the bacterial growth, which at the concentration of 3.54 µg/mL leads to a bactericidal effect. To explain this surprising effect, we collected SEM images of the bacterial culture which was administered the AuNP solution with a concentration of 3.54 µg/mL, and irradiated with the low-power laser. We observed the formation of nanoparticle clusters on the bacterial wall. These clusters are not formed in the absence of bacteria except using high power lasers. These clusters are responsible for the low-power-induced photothermal effect, and their formation is catalyzed by the bacterial wall.

In conclusion, the results show that these nanoparticles constitute a proof-of-concept of a photothermal system able to activate only in the presence of the pathogen, and to have a bactericidal effect even at low concentrations.

## Figures and Tables

**Table 1:** *E. coli* CFU/mL exposed to different concentrations of AuNP, with and without irradiation, and the percentage of growth inhibition after 24 h of growth in incubator. No changes were recorded for the control (*E. coli* CFU/mL 2.1 × 10^8^ with and without irradiation). The data are reported as the average of three determinations made in duplicate ± standard deviation.

	Dark Condition		Under Irradiation	
AuNP Concentration	*E. coli* (CFU/mL)	% Growth Inhibition	*E. coli* (CFU/mL)	% Growth Inhibition
0.26 µg/mL	2.1 × 10^8^ ± 0.179 × 10^8^	0	2.12 × 10^8^ ± 0.133 × 10^8^	0
0.39 µg/mL	2.08 × 10^8^ ± 0.227 × 10^8^	0	2.08 × 10^8^ ± 0.232 × 10^8^	0
1.56 µg/mL	1.8 × 10^8^ ± 0.145 × 10^8^	−15%	0.977 × 10^8^ ± 0.117 × 10^8^	−53%
3.54 µg/mL	1.23 × 10^8^ ± 0.232 × 10^8^	−46%	2.1 × 10^6^ ± 0.219 × 10^6^	−99%

## Supplementary Materials

The following supporting information can be downloaded at: https://www.mdpi.com/article/10.3390/nano13040746/s1, Figure S1: DLS measurement; Figure S2: Thermal photo of AuNS@PEG-SH water solution (3.54 µg/mL) irradiated with a laser source (λ = 532 nm, 60 mW). Room temperature 19 °C; Figure S3: Thermal photo of AuNS@PEG-SH-saturated water solution irradiated with a laser source (λ = 532 nm, 60 mW). Room temperature 19 °C; Figure S4: Thermal photo of AuNS@PEG-SH water solution (3.54 µg/mL) irradiated with a laser source (λ = 532 nm, 1500 mW). Room temperature 19 °C; Figure S5: Thermal photo of AuNS@PEG-SH-saturated water solution irradiated with a laser source (λ = 532 nm, 1500 mW). Room temperature 19 °C. Table S1. Results of the original experiments with counting colonies.
