# Supplementary material for: Light-Induced Clusterization of Gold Nanoparticles: A New Photo-Triggered Antibacterial against E. coli Proliferation
Source: Nanomaterials (Basel). 2023 Feb 16;13(4):746. doi: 10.3390/nano13040746 (PMC9967119; doi:10.3390/nano13040746)
Supplement: Supplementary file 1 [file nanomaterials-13-00746-s001.zip › nanomaterials-2218544-supplementary.pdf]

Supplementary information

Light-induced clusterization of Gold Nanoparticles: a new Pathogen-responsive candidate against *E. Coli* Infection

[1,2]Angela Candreva, [2]Renata De Rose, [3]Ida Daniela Perrotta, [2,4] Alexa Guglielmelli and [1,2]Massimo La Deda*

[1] Department of Chemistry and Chemical Technologies, University of Calabria, 87036 Rende, CS, Italy; [2] CNR-NANOTEC, Institute of Nanotechnology U.O.S, Cosenza, 87036 Rende, CS, Italy; [3] Department of Biology, Ecology and Earth Sciences, Centre for Microscopy and Microanalysis (CM2), University of Calabria, 87036 Rende, CS, Italy; [4]Department of Physics, NLHT-Lab, University of Calabria, 87036 Rende (CS), Italy

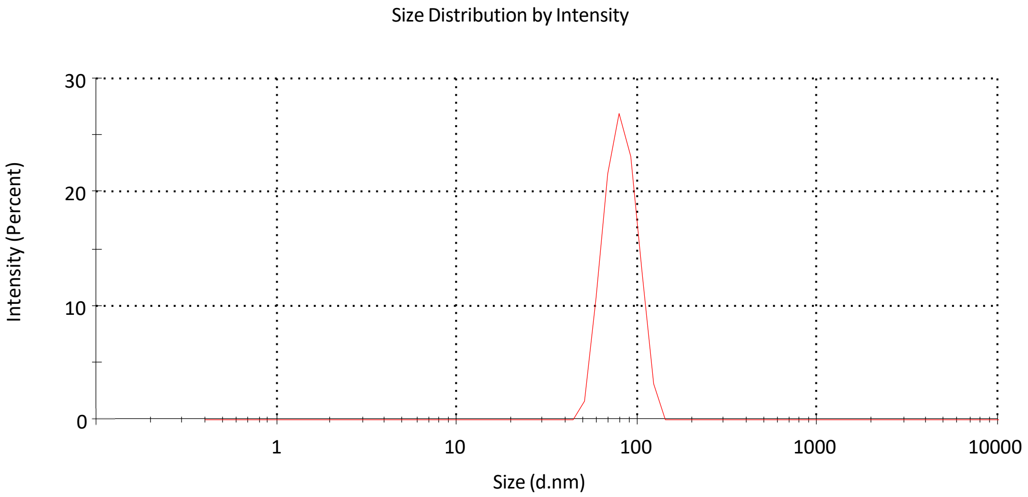

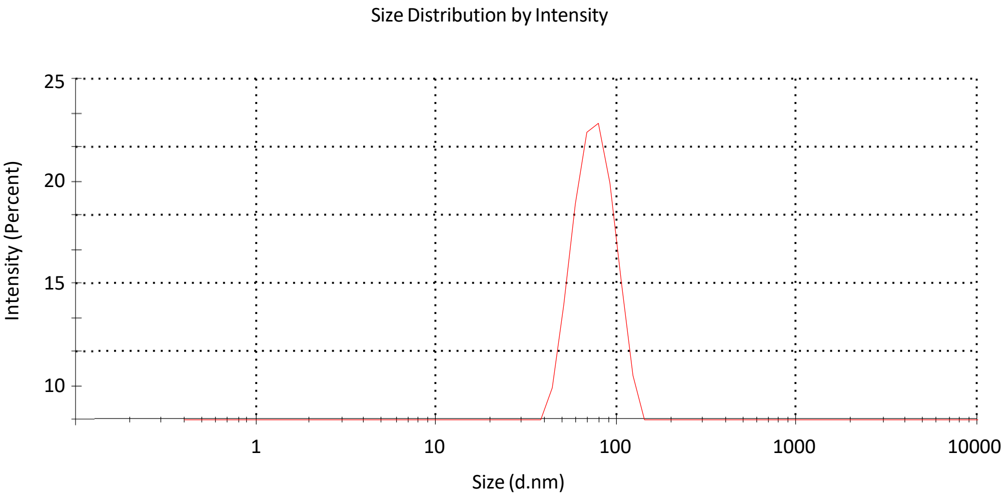

Fig. S1 DLS measurement AuNS@PEG in water (on the top); DLS measurement AuNS@PEG in PBS (on the bottom).

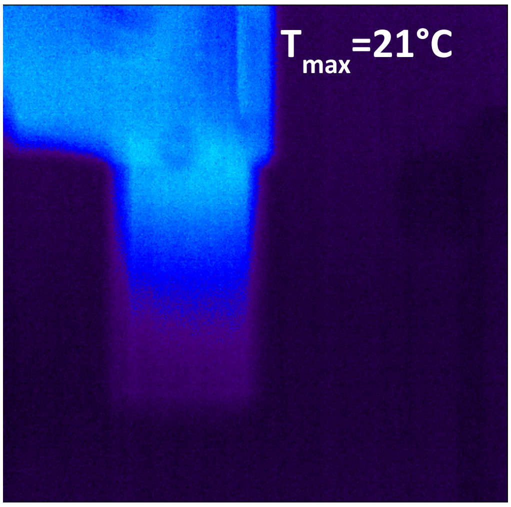

Fig.S2 Thermal photo of AuNS@PEG-SH water solution (3.54 μg/mL) irradiated with a laser source for 5 min (λ= 532 nm, 60 mW). Room temperature 19°C.

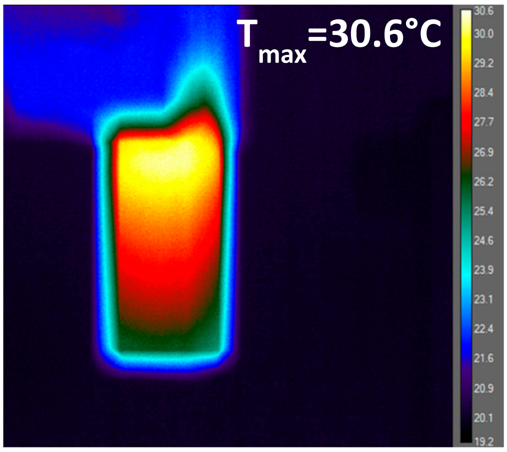

Fig.S3 Thermal photo of AuNS@PEG-SH saturated water solution irradiated with a laser source for 5 min(λ= 532 nm, 60 mW). Room temperature 19°C.

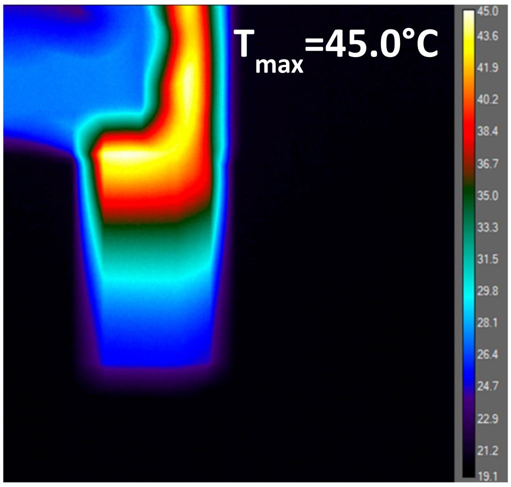

Fig.S4 Thermal photo of AuNS@PEG-SH water solution (3.54 µg/mL) irradiated with a laser source for 5 min (λ= 532 nm, 1500 mW). Room temperature 19°C.

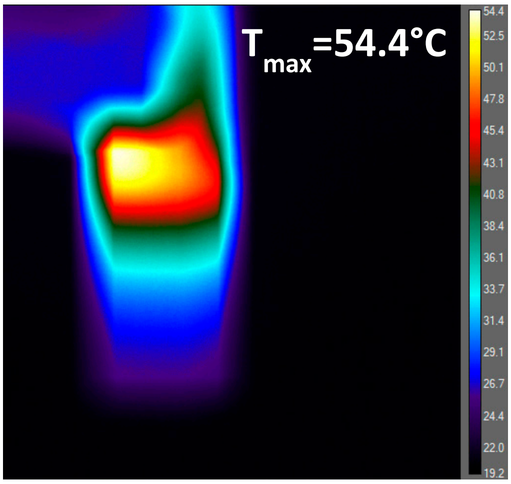

Fig.S5 Thermal photo of AuNS@PEG-SH saturated water solution irradiated with a laser source for 5 min (λ= 532 nm, 1500 mW). Room temperature 19°C.

Table S1. Each experiment was performed in duplicate and repeated 3 times. From this data population, the average and the standard deviation of the bacterial counts have been obtained and reported.

	Dark condition		Under irradiation	
AuNP concentration	*E. coli* (CFU/mL)	%growth inhibition	*E. coli* (CFU/mL)	%growth inhibition
0.26 µg/mL	$1.9x10^8$; $2.3x10^8$<br>$2.1x10^8$; $2.1x10^8$<br>$2.3x10^8$; $1.9x10^8$<br>*Average* $2.1x10^8$<br>*Standard deviation* $0.179x10^8$	0	$2.2x10^8$; $2.0x10^8$<br>$2.3x10^8$; $2.0x10^8$<br>$2.0x10^8$; $2.2x10^8$<br>*Average* $2.12x10^8$<br>*Standard deviation* $0.133x10^8$	0
0.39 µg/mL	$1.8x10^8$; $2.2x10^8$<br>$2.2x10^8$; $2.4x10^8$<br>$1.95x10^8$; $1.9x10^8$<br>*Average* $2.1x10^8$<br>*Standard deviation* $0.227x10^8$	0	$1.85x10^8$; $2.0x10^8$<br>$2.35x10^8$; $2.4x10^8$<br>$1.95x10^8$; $1.95x10^8$<br>*Average* $2.08x10^8$<br>*Standard deviation* $0.232x10^8$	0
1.56 µg/mL	$2.0x10^8$; $1.6x10^8$<br>$1.85x10^8$; $1.75x10^8$<br>$1.9x10^8$; $1.7x10^8$<br>*Average* $1.8x10^8$<br>*Standard deviation* $0.145x10^8$	-15%	$0.96x10^8$; $1.1x10^8$<br>$0.8x10^8$; $1.0x10^8$<br>$0.9x10^8$; $1.1x10^8$<br>*Average* $0.977x10^8$<br>*Standard deviation* $0.117x10^8$	-53%
3.54 µg/mL	$1.4x10^8$; $1.0x10^8$<br>$0,97x10^8$; $1.5x10^8$<br>$1.1x10^8$; $1.4x10^8$<br>*Average* $1.23x10^8$<br>*Standard deviation* $0.232x10^8$	-46%	$1.9x10^6$; $1.8x10^6$<br>$2.2x10^6$; $2.1x10^6$<br>$2.2x10^6$; $2,4x10^6$<br>*Average* $2.1x10^6$<br>*Standard deviation* $0.219x10^6$	-99%
